# Supplementary material for: The PB2 Polymerase Host Adaptation Substitutions Prime Avian Indonesia Sub Clade 2.1 H5N1 Viruses for Infecting Humans
Source: Viruses. 2019 Mar 22;11(3):292. doi: 10.3390/v11030292 (PMC6480796; doi:10.3390/v11030292)
Supplement: Supplementary file 1 [file viruses-11-00292-s001.pdf]

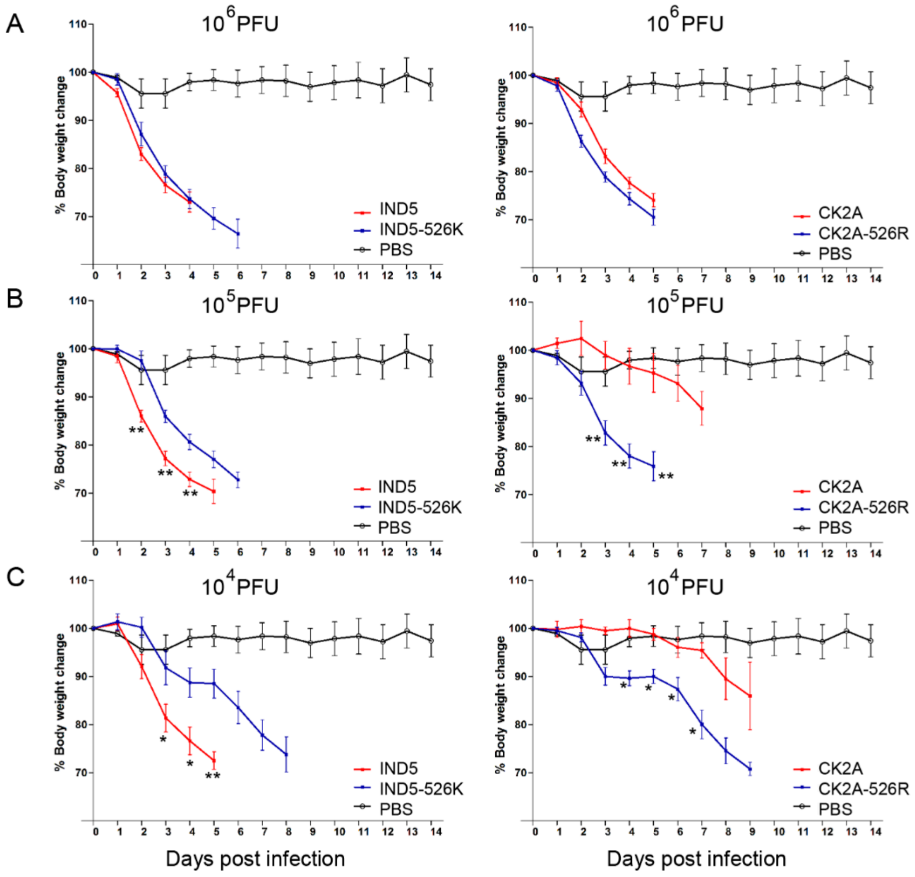

Figure S1. H5N1 virus infection in mice.

Groups of four or six BALB/c mice, aged 4-6 weeks, were intranasally inoculated with 106 (A), 105 (B) or 104 (C) PFU of wild type IND5 or CK2A, or 526K (IND5-526K) or 526R (CK2A-526R) mutant viruses, in 25ul PBS. Body weight and survival were monitored daily for 14 days after infection. Error bars represent data of standard deviation from different virus-infected mouse. Statistical significance was analyzed by one-way ANOVA or Student's t-test ***p<0.001, **p<0.01 and *p<0.05
